# Risk Perception and Protective Behaviors During the Rise of the COVID-19 Outbreak in Italy

**DOI:** 10.3389/fpsyg.2020.577331

**Published:** 2021-01-13

**Authors:** Lucia Savadori, Marco Lauriola

**Affiliations:** ^1^Department of Economics and Management, University of Trento, Trento, Italy; ^2^Department of Social and Developmental Psychology, Sapienza University of Rome, Rome, Italy

**Keywords:** risk perception, cultural worldviews, COVID-19, coronavirus, affect, social norms, health behavior, SARS-CoV-2

## Abstract

Risk perception is important in determining health-protective behavior. During the rise of the COVID-19 epidemic, we tested a comprehensive structural equation model of risk perception to explain adherence to protective behaviors in a crisis context using a survey of 572 Italian citizens. We identified two categories of protective behaviors, labeled promoting hygiene and cleaning, and avoiding social closeness. Social norms and risk perceptions were the more proximal antecedents of both categories. Cultural worldviews, affect, and experience of COVID-19 were the more distal predictors. Promoting hygiene and cleaning was triggered by the negative affective attitude toward coronavirus and mediated by an affective appraisal of risk. The deliberate dimension of risk perception (perceived likelihood) predicted only avoiding social closeness. Social norms predicted both types of behaviors and mediated the relations of cultural worldviews. Individualism (vs. communitarianism), more than hierarchy (vs. egalitarianism), shaped the affective evaluation of coronavirus. The model was an acceptable fit to the data and accounted for 20% and 29% of the variance in promoting hygiene and cleaning, and avoiding social closeness, respectively. The findings were robust to the effect of sociodemographic factors (age, gender, education, socioeconomic status, and zone of the country). Taken together, our findings confirmed the empirical distinction between affective and deliberate processes in risk perception, supported the validity of the affect heuristic, and highlighted the role of social norms as an account for why individualistic people were less likely to follow the prescribed health-protective behaviors. Implications for risk communication are discussed.

## Introduction

Individual behavior and risk perception are two interrelated aspects of a disease outbreak. Higher perceived risk can increase an individual’s adherence to preventive measures (e.g., Brewer et al., 2007) and control the spread of the outbreak. It is important to gain insights into the factors predicting risk perception and their impact on the adherence to protective measures during the COVID-19 pandemic.

The present research was conducted in a crisis scenario. We ran a survey in Italy on 13 March 2020, 2 days after the government issued the national lockdown on 11 March (lasting for 54 days, until 4 May). The total infected cases were 17,660, and the death toll was 1,266 out of 60 million inhabitants. The COVID-19 outbreak was just at the beginning, and the north of the country was mostly involved. Two months later, the total positive cases were 221,216, the death toll had grown to 30,911, and COVID cases were diagnosed nationwide. Our data portray a period in which the disease infection was spreading, the emergency was rising, and the attention of the media and the entire population was overly focused on the hazard.

The first non-imported COVID-19 case in Italy was discovered on 21 February 2020 in Codogno, a small town in the Lombardy region, in the north of the country. From that first hotbed, it soon became clear that the disease could spread to nearby towns and regions. On 4 March, 12 days after the first case, the Italian government closed the schools and the universities. The confirmed positive cases were only 2,700. Four days later, on 8 March, a decree was issued to isolate the Lombardy region and 14 nearby provinces. Measures to contain the infection were envisaged considering the epidemiological dynamics developed in the earlier days, including “avoiding any movement of natural persons entering and leaving the territories […], and within the same territories, except for movements motivated by proven work needs, or cases of necessity, or movements for reasons of health. Return to your home, home or residence is allowed” (Decree of the Presidency of the Council of Ministers, 8 March 2020).

Massive media coverage was given to these measures. But a national lockdown was not issued until 11 March. Common retail businesses, educational activities, and catering services were suspended, and gatherings of people in public places or places open to the public were prohibited. To face the emergency, the government gave precise instructions to the citizens: before lockdown, a series of health-protective actions that citizens had to follow were already issued and promoted in schools, universities, and public offices (Decree of the Presidency of the Council of Ministers 1 March 2020)^1^; town mayors and trade associations ensured the largest diffusion of the recommended actions in commercial buildings (from pharmacies to supermarkets).

According to health behavior models, adherence to recommended safety practices depends on individuals’ risk perception. For example, the intention to get vaccinated against diseases is greater among individuals perceiving the probability of contracting that disease as higher (Brewer et al., 2007). Risk perception is central to many models that explain behaviors related to health-related choices (e.g., Health Belief Model; Rosenstock, 1974). Also, major behavioral models such as the Theory of Reasoned Action (Fishbein and Ajzenet al., 1975), the Theory of Planned Behavior (Ajzen, 1991), and the Subjective Expected Utility Theory (Edwards, 1954; Sutton, 1987; Ronis, 1992) argue that the probability and the magnitude of a potential hazard (risk perception) are crucial factors in shaping risk behavior.

Although a relationship between risk perception and protective behavior has often been found, its strength has been questioned (Brewer et al., 2007). The purpose of our study is to provide a systematic and theoretically integrated overview of the main determinants of COVID-19 risk perception and its relationship with the recommended protective behaviors. A comprehensive model is proposed and the explanatory power of the model is empirically tested on a national sample of the Italian population during the COVID-19 emergency outbreak using a set of highly reliable measurement constructs. Since decisions are not made in a social vacuum, this study further examines to what extent COVID-19 risk perception is explained by individual-level (i.e., experience, affect, risk perception) and social-level factors (i.e., cultural worldviews and social norms).

## The Present Research

### Risk Perception

Early studies of risk perception used a variety of psychometric methods to produce quantitative measures of perceived risk and perceived benefits. This general approach, known as the psychometric paradigm (Fischhoff et al., 1978; Lichtenstein et al., 1978), led to mapping several hazards onto a bi-dimensional diagram derived from a factor analysis of nine dimensions of risk (e.g., controllability, dreadfulness, etc.). The two factors reflected the degree to which the risk from a particular hazard was known and how much that hazard evoked feelings of dread. Research showed that laypeople’s perceptions of risk were related to where each hazard was located within this bi-dimensional space (Slovic, 1987). The findings from the psychometric studies evolved in the proposal that there are two fundamental ways in which humans perceive and act on risk (Slovic et al., 2004). The first, called “risk as feelings,” describes one’s instinctive and intuitive reactions to threat. The second, called “risk as analysis,” is based on logic, reason, and deliberative processes. Reliance on risk as feelings is described as the affect heuristic (Finucane et al., 2000a). Reliance on feelings is faster, effortless, and more efficient than reliance on analysis to navigate in a complex, uncertain, or dangerous environment (Epstein, 1994).

Some scholars proposed a tripartite model of risk perceptions including deliberative, affective, and experiential aspects (Ferrer et al., 2016). The deliberative risk perception corresponds to the perceived likelihood of incurring a negative event. The feeling component of risk perception has been further divided into experiential and affective aspects deemed to be distinct factors useful to explaining individual behavior in the health domain (Ferrer et al., 2016; Kaufman et al., 2020). In particular, the experiential risk perception is the “gut” feeling of being vulnerable to the risk and is assessed using items such as “My first reaction when I hear of someone getting lung cancer is ‘that could be me someday”’ (Ferrer and Klein, 2015; Ferrer et al., 2016). Affective risk perception is the feeling experienced when thinking about a hazard and responds to questions such as “How worried are you about getting the flu this season?” (Ferrer et al., 2016, 2018; Kaufman et al., 2020).

Previous research has shown that the affective component is the strongest predictor of protection motivation across a variety of hazards (Hay et al., 2006; Janssen et al., 2014; Ferrer et al., 2018), and interventions targeting the affective risk perception are the most successful (Sheeran et al., 2014). Also, the experiential risk perception seems to predict a variety of protective behaviors better than perceived likelihood (Weinstein et al., 2007; Janssen et al., 2011). However, research suggests that affective and emotional processes interact with reason-based analysis in all normal thinking processes and are essential to rationality (Damasio, 1994).

The present study adopted a comprehensive approach. We not only measured all three dimensions of risk perception but also included a set of general standard questions related to risk perception as a societal issue (see Table 1). Following Kaufman et al. (2020), we also included two items that make the risk assessment contingent on undertaking risk behavior or protective behavior. Conditional risk perception reflected one’s belief of getting hurt if one does not follow safety rules (e.g., if you do not follow the recommendations, what are your chances of getting coronavirus?). Recent studies have strongly recommended including this measure to improve the prediction of protective behavior (Taylor and Snyder, 2017; Kaufman et al., 2020). By using multiple indicators, we aimed to assess separate aspects of risk perception, each of which might be in a different predictive relation with health-protective behavior.

**TABLE 1:** Characteristics of COVID-19 affective attitude and risk perception items.

Characteristic	Index	Description	Source	Item
Affective Attitude	Affect	The holistic affective reaction associated with a hazard	van der Linden, 2015	I see coronavirus as something that is … ^2^ Overall, I feel that coronavirus is…^3^ To me, coronavirus is…^1^
			Hadjichristidis et al., 2015	If I think of coronavirus I feel mostly ………. feelings ^1^
Risk perceptions	Feelings of risk	Affective risk perception	Ferrer et al., 2018 Kaufman et al., 2020	When you think about coronavirus, to what extent do you feel fearful?^4^ How worried are you about coronavirus?^4^
		General risk perception	Peters et al., 2011	In general, how risky do you think coronavirus is? ^4^
			Finucane et al., 2000a	In general, how risky do you consider coronavirus to be to Italian society as a whole? ^4^
			Dohmen et al., 2011	How much risk do you believe that coronavirus poses to human health, safety or prosperity? ^4^
		Experiential risk perception	Ferrer et al., 2018 Kaufman et al., 2020	To what extent do you feel vulnerable to coronavirus?^4^ When you hear of someone having coronavirus, to what extent your first reaction is ‘that could be me someday’?^4^
	Risk Analysis	Perceived likelihood	Kaufman et al., 2020	How likely do you think it is that you will get coronavirus?^4^
		Conditional risk perception	Dillard et al., 2012	If you did not follow the recommendations to reduce the infection issued by the President of the Council of Ministers, how much do you think it would be likely for you to contract the coronavirus?^4^
			Kaufman et al., 2020	If you continue to adopt the usual lifestyle you have led up to now, how likely would you be to get coronavirus?^4^

*Response Scales: ^1^Very negative, fairly negative, slightly negative, neither negative nor positive, slightly positive, fairly positive, very positive. ^2^Extremely unpleasant, very unpleasant, quite unpleasant, slightly unpleasant, neither unpleasant nor pleasant, slightly pleasant, quite pleasant, very pleasant, extremely pleasant. ^3^A very bad thing, a pretty bad thing, a slightly bad thing, neither a bad thing nor a good thing, a slightly good thing, a pretty good thing, a very good thing. ^4^Not at all, Slightly, Moderately, Very, Extremely.*

### Affective Attitude

Among the determinants of risk perception, a dominant conception suggests that it largely depends on intuitive and experiential processes, guided by emotional and affective factors, rather than conscious and analytical processes deliberately implemented by the perceiver (Finucane et al., 2000a; Slovic et al., 2004). In this conception, risk perception originates from a general affective assessment, from which the risk and benefit judgments both derive. This assessment has been termed “affect” and means the specific quality of “goodness” or “badness” (Slovic et al., 2004). According to this view, it is not so much the analytical thoughts about potential pros and cons that determine the perception of risk, but a general affective attitude toward the object of risk perception. For example, the perceived risks and benefits associated with nuclear power are best predicted by people’s beliefs about the extent to which nuclear power is good or bad, positive or negative, pleasant or unpleasant (Slovic et al., 2004).

The affect heuristic assumes that to derive perceived risk about a hazard, we intuitively and involuntarily hinge upon the affective attitude for that hazard (e.g., how good or bad it is). This affective value summarizes into a simple evaluation all of our direct and indirect experiences with that hazard. This is used in subsequent evaluation and decisions about risk. In particular, if the affective attitude is positive (i.e., I like it), then risks are judged low and benefits high; conversely, if the affective attitude is negative (i.e., I dislike it), the risks are judged high and benefits low. In this heuristic model, therefore, the affective attitude predicts risk perception. This process has received plenty of empirical support in both the health (Peters et al., 2006a) and non-health domains, such as in the perception of risk for a wide range of different hazards (Hadjichristidis et al., 2015; Skagerlund et al., 2020), the expected returns of a risky financial asset (Statman et al., 2008), the subjective riskiness of a gamble (Mukherjee, 2010), and flooding risk perception (Keller et al., 2006).

**FIGURE 1:**
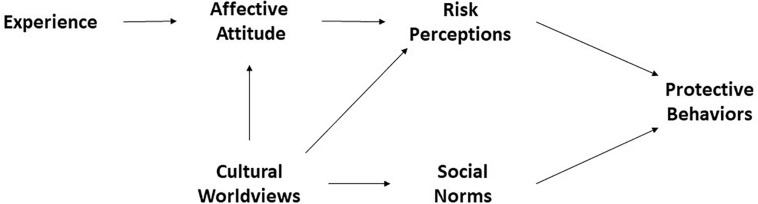
The COVID-19 risk perception and protective behavior model (primary theoretical model).

Under the affect heuristic hypothesis, the current study measured the affective attitude toward coronavirus in a holistic way (Table 1). We hypothesized that the affective attitude would significantly influence an individual’s risk perception. In other words, the greater the extent to which coronavirus is viewed negatively, the more it is viewed as risky (Figure 1).

Relying on affect is the preferred strategy when people are under stress, knowledge is low, decisions must be made quickly, and there is no room for mistakes (Finucane et al., 2000a). Coherently with this assumption, the present research measured the affective attitude toward coronavirus during the COVID-19 pandemic emergency, when contagion risk from a new virus was rising and its consequences were still unknown. Under these circumstances, we expected people’s judgments of risk to strongly depend on their affective attitude.

### Direct and Indirect Experience

Learning processes through direct (Leventhal et al., 1965; Öhman and Mineka, 2001) or indirect experience (Tversky and Kahneman, 1973; Lichtenstein et al., 1978) with a hazard are the core elements of the affective value used in the affect heuristic (Slovic et al., 2007). Damasio, for example, maintained that a lifetime of learning leads mental images to become marked by positive and negative feelings linked directly or indirectly to somatic or bodily states (Bechara et al., 1996). When an image is associated with a negative marker, it sounds like an alarm. People’s perceived likelihood of an event also depends on the availability with which examples of that event arise in memory (Tversky and Kahneman, 1973), which is strongly determined by personal experience but also by indirect experience of the event, for example, through the mass media (Lichtenstein et al., 1978). Affective reactions associated with coronavirus will, therefore, follow these rules. Direct and indirect experience with the virus can increase the strength of the affective attitude associated with COVID-19. We expected that knowing a person who has been hospitalized for coronavirus would increase one’s negative attitude toward the virus and the associated perceived risk (Figure 1).

### Cultural Worldviews and Risk Perception

How can one have an emotional attitude toward something one does not know? When there is no experience of a hazard, and little personal and scientific knowledge is available, it is difficult to attach a clear evaluative value to it. To understand how dangerous the coronavirus is, people may compare it with seasonal influenza or common pneumonia, but again, COVID-19 remains an unknown threat (even more so at the time we conducted this study). In the face of this new threat, it is reasonable to believe that people’s attitudes and perceptions could be fed by other factors, rather than experience, such as social and cultural factors.

Social, political, and cultural factors play a significant role in risk perception. Among these, cultural worldviews of *hierarchy–egalitarianism* (hierarchy) and *individualism–communitarianism* (individualism) have gained noticeable importance in explaining risk perception and individual attitudes (Peters and Slovic, 1996; Kahan et al., 2011, 2012; Drummond and Fischhoff, 2017). According to Dake (1991), worldviews are general attitudes that people have toward the world and its social organization. In particular, hierarchy reflects attitudes toward the social systems that link authority to a stratified social role based on explicit characteristics such as gender, race, and class (e.g., “We have gone too far in pushing equal rights in this country”). On the other side, egalitarianism emphasizes equal distribution of wealth as a priority (e.g., “We need to dramatically reduce inequalities between the rich and the poor, whites and people of color, and men and women”). Individualism reflects attitudes toward the social systems that reveal the expectation that individuals guarantee their well-being without assistance or interference from the government and the society (e.g., “The government should stop telling people how to live their lives”). Conversely, communitarianism assigns to society the obligation to guarantee the collective well-being and the power to prevail over the interest of the individual (e.g., “Sometimes government needs to make laws that keep people from hurting themselves”).

These worldviews can shape the content of an individual’s imagery and its affective evaluation and guide behavior and choices in controversial matters, such as policies to prevent climate change (Kahan et al., 2012; Drummond and Fischhoff, 2017). According to the cultural theory of risk (Douglas and Wildavsky, 1982; Dake, 1991), worldviews can also guide risk perceptions. For example, egalitarian individuals perceived higher risk and were more worried about nuclear power plants than hierarchical ones, who appraised a lower risk and had more favorable attitudes (Peters and Slovic, 1996). Both worldviews and affective evaluations acted as “dispositions” that guided and helped people to appraise risks and respond to threats. Notably, expert judgments are also influenced by worldviews and affective attitudes (Kunreuther and Slovic, 1996; Slovic, 1999; Savadori et al., 2004).

The current study measured both hierarchy–egalitarianism and individualism–communitarianism, under the hypothesis that hierarchical and individualistic views significantly influence one’s affective attitude. In particular, we expect that the more an individual holds hierarchical and individualistic views, the less negatively he/she appraises the coronavirus, with a subsequent reduction in risk perceptions (see Figure 1).

### Social Norms and Protective Behavior

Citizens’ safety during a pandemic depends on the extent to which they comply with the prescribed protective measures. Social norms are a fundamental construct in behavior change (Cialdini et al., 1990, 1991; Ostrom, 2000; Schultz et al., 2007; Goldstein et al., 2008; Allcott, 2011). Some scholars underscore the difference between descriptive and prescriptive (or injunctive) norms (Borsari and Carey, 2003; Rimal and Real, 2003b, 2005; Lapinski and Rimal, 2005). Descriptive norms reflect the subjective perception of what others do, whereas prescriptive norms are the belief about what one is expected to do (Cialdini et al., 1990, 1991; Cialdini, 2007). Both types of norms are deemed important in determining behavior. For example, according to the Theory of Normative Social Behavior, prescriptive norms can moderate the influence of descriptive norms on behavior (Rimal and Real, 2003b, 2005). For example, seeing that other people smoke (descriptive norms) can increase my tendency to smoke, but it depends on whether I think it is acceptable for me to smoke (prescriptive norms). Descriptive norms and prescriptive norms have been successfully applied to promote hand-washing (Pedersen et al., 1986; Munger and Harris, 1989; Lapinski et al., 2013) and health behaviors (Curtis et al., 2011). Social norms have been suggested to be potential triggers of protective actions during the COVID-19 pandemic (Andrews et al., 2020; Bavel et al., 2020).

Given that social norms are likely to influence behavior, the present study measured both descriptive and prescriptive norms associated with undertaking protective actions against coronavirus. The hypothesis was that the more one thinks that significant others are acting to prevent coronavirus contagion (descriptive), and the more one feels socially pressured to reduce the risk of contagion (prescriptive), the more he/she will comply with protective measures. An additional hypothesis is that social norms mediate the relationship between cultural worldviews and undertaking protective actions, especially those prescribed from an authority to contrast the spread of the epidemic. We have relied on the idea that the most egalitarian/communitarian individuals are more likely to adhere to such behaviors, to the extent that they feel compelled to comply with social norms when it comes to pursuing a common good.

### The Model

The constructs examined in our literature review and our hypotheses regarding their relationships can be summarized in a general model, which we aimed to test by examining how well it could account for the data collected during the coronavirus epidemic in Italy. According to the “affect heuristic,” we assign a central role to affective attitude, informed by experience, in shaping risk perceptions. Risk perceptions, both affective and deliberate, are the more proximal antecedents of protective actions. In keeping with the cultural construction of risk, we consider the cultural worldviews as predictors of affective attitude and risk perceptions. Lastly, the model incorporated the concept that perceived social norms, influenced by cultural worldviews, could shape health behavior.

We do not intend to offer a definitive description, nor is the range of predictors intended to be overall complete. We aimed to provide an operational framework to better incorporate individual and social factors into the understanding of coronavirus risk perception and risk protective behavior. This study aims to discuss the complex interplay among social, cultural, and affective factors in determining risk perception and risk protection during the COVID-19 outbreak.

## Materials and Methods

### Sample

The study is based on a nationwide sample of the Italian population (*N* = 572) comprised of individuals who subscribed to www.prolific.ac, a commercial crowdfunding platform for the recruitment of subjects for survey research. The sample was composed of 54% male and 46% female respondents. The age of participants ranged between 18 and 45 years, with an average of 26 years (*SD* = 6.4). The majority of the sample (46%) lived in the north of the country, 26% in the center, and 28% in the south or islands, reflecting the distribution of the national population density. The distribution of education was as follows: 4%, 51%, 42%, and 3% for middle school, high school, university, and Ph.D. levels, respectively. The socioeconomic status of the participants was categorized as low (10%), medium (54%), or high (36%).

### Materials and Procedure

The survey was administered online on 13 March 2020. Each participant was rewarded £ 1.41. The completion time was about 11 min, on average. The survey was anonymous. The procedure was approved by the Ethics Committee for Experimentation with the Human Being of the University of Trento (protocol no. 2020-020). The order of the sections measuring risk perception, affective attitude, and social norms was randomized across participants, whereas the sections measuring behaviors, experience, worldviews, and sociodemographic information were presented to all participants after the previous three and in a fixed order. The items were randomized within each section.

### Measures

#### Protective Behaviors

We used 13 items to assess self-reported compliance with protective behaviors recommended by the Italian Government to prevent the spread of coronavirus infection (see Table 2). We asked the participants to report how often they have adhered to each behavior. The exact wording was: “Think about the behaviors you are having these days. How much are you taking each of the following health prevention measures?” We collected responses using a six-point scale ranging from *never* (1) to *always* (6). The items were preliminarily submitted to a principal component analysis (see Supplementary Materials S1–S3). The inspection of initial eigenvalues, corroborated by parallel analysis, suggested retaining two factors that were obliquely rotated (PROMAX). The first factor (23% of explained variance after rotation) loaded on items primarily associated with social distancing (e.g., maintaining an interpersonal distance of at least one meter) and inhibition of habitual behaviors (e.g., avoid hugs and handshakes with your acquaintances). The second factor (22% of explained variance after rotation) loaded on items describing one’s compliance with hand hygiene prescriptions (e.g., wash your hands often) and active protection behaviors (e.g., clean surfaces with chlorine- or alcohol-based disinfectants). We computed two composite scores, with a higher value reflecting a higher tendency to *promote hygiene and cleaning* (α = 0.77) and *avoiding social closeness* (α = 0.73), respectively.

**TABLE 2:** Recommendations used in the study to measure COVID-19 protective behaviors.

List of recommendations
(1) Wash your hands often
(2) Use hydroalcoholic solutions for hand washing if made available in public places, gyms, supermarkets, pharmacies, and other meeting places
(3) Avoid close contact with people you know who suffer from acute respiratory infections
(4) Avoid hugs and handshakes with your acquaintances
(5) Avoid hugs and handshakes with your close relatives
(6) Maintaining, in social contacts, an interpersonal distance of at least one meter
(7) Sneezing and/or coughing in a tissue or elbow, avoiding contact of the hands with respiratory secretions
(8) Avoid the promiscuous use of bottles and glasses, especially during sports
(9) Do not touch your eyes, nose, and mouth with your hands
(10) Cover your mouth and nose if you sneeze or cough
(11) Do not take antiviral drugs and antibiotics unless prescribed by your doctor
(12) Clean the surfaces with chlorine or alcohol-based disinfectants
(13) Use the face mask if you suspect you are ill or if you are caring for sick people

#### Risk Perception

In keeping with Kaufman et al. (2020), we used multiple items to assess risk perception (see Table 1). Ten items covered affective, experiential, deliberate, general, and conditional risk dimensions. The affective items asked to what extent the participant felt fearful thinking about coronavirus, and how worried they were about getting coronavirus. Two experiential items asked about perceived vulnerability toward coronavirus. Three items asked about perceived risk in general, for the Italian society, and for human health, safety, and prosperity. We also asked how likely it was for participants to be infected with the coronavirus, both in general and conditional to the fact that they would not follow the recommendations to reduce the infection and instead continued to behave as before. A preliminary principal component analysis showed that two correlated factors were appropriate to represent the underlying structure of risk perceptions (see Supplementary Materials S4–S6). The first factor (38% of explained variance after rotation) loaded on items describing the affective, experiential, and general risk perceptions. The second factor (15% of explained variance after rotation) was loaded on items primarily associated with the perceived likelihood of getting the coronavirus. These factors resembled the distinction between the *affective/experiential* and *analytic systems* (Slovic et al., 2004). Two composite scores were computed, with a higher score reflecting a higher tendency to rely on *feelings of risk* (α = 0.88) and *risk analysis* (α = 0.64) to inform decision making, respectively.

#### Affective Attitude

Drawing on items used in previous work (Hadjichristidis et al., 2015; van der Linden, 2015) and following Peters and Slovic (2007), we used four items to assess the holistic affective reaction associated with coronavirus (see Table 1). All the questions asked respondents to rate coronavirus using bipolar adjectives (very negative – very positive, extremely unpleasant – extremely pleasant, a very bad thing – a very good thing). We obtained a reliable total score for *affective attitude* (α = 0.86), with lower ratings reflecting a more negative affective evaluation of coronavirus.

#### Experience

Following Lichtenstein et al. (1978), we used two ratings of indirect experience by asking participants to report how often they had heard about coronavirus via the media (newspapers, magazines, radio, television, Internet, etc.) as a cause of death and as a cause of suffering (but not death). Ratings were made on a six-point scale ranging from *never* (1) to *always* (6). Direct experience of coronavirus as a cause of death was measured by a multiple-choice item: At least one close friend or relative has died from coronavirus (coded as 2); someone I know (other than a close friend or relative) has died from coronavirus (coded as 1); no one I know has died from coronavirus (coded as 0). Likewise, direct experience of coronavirus as a cause of suffering was measured replicating the same item but replacing “died” with “suffered (but not died).” We obtained two summated ratings, one for direct experience (ranging from 0 to 4) and another for indirect experience (ranging from 2 to 12).

#### Cultural Worldviews

Drawing on previous work by Kahan et al. (2011), we assessed *hierarchy* and *individualism* using the short-form version of the cultural worldview scale. The scale includes 12 items that tap into worldviews along two cross-cutting dimensions: *hierarchy–egalitarianism* and *individualism–communitarianism*. For all items, participants indicated agreement or disagreement on a seven-point scale (1 = completely disagree; 7 = completely agree). Reliable *hierarchy–egalitarian* (α = 0.81) and *individualism–communitarian* (α = 0.71) scales were obtained.

#### Social Norms

Drawing on items developed by van der Linden (2015), descriptive norms were measured asking respondents to answer three questions about how likely they think it is that important referent others are taking personal action to help tackle coronavirus on a seven-point Likert scale (1 = strongly disagree; 7 = strongly agree). The wording was as follows: “Most people who are important to me are personally doing something to help reduce coronavirus risk”; “Most people I care about do their best to help slow down coronavirus infection”; “People close to me are taking personal action to reduce the risk of coronavirus.” Prescriptive norms were measured by asking respondents four questions about the extent to which they feel socially pressured to personally help reduce the risk of coronavirus. The wording was as follows: “Overall, I am expected to do my best to help reduce coronavirus risk”; “The people who are important to me would support me if I decided to help reduce the risk of coronavirus”; “The people whose opinion I value think I should act personally to reduce the risk of coronavirus”; “I feel that helping to cope with coronavirus risk is something that is NOT expected of me.” Although previous studies used separate scores for descriptive and prescriptive norms, in the present study the two composite scores were highly inter-correlated (*r* = 0.72), and a principal component analysis of the seven items yielded a unidimensional structure, accounting for 59% of the variance in social norms items (see Supplementary Materials S7–S9). As a result, we calculated a single composite score that was highly reliable (α = 0.86).

#### Sociodemographic Characteristics

A range of sociodemographic information was collected. Each participant was asked about the place of residence, asking to report where he/she lived when completing the survey. This information was further recoded into three categories (1 = north, 2 = center, 3 = south and islands). The level of education was assessed by asking, “Which is the highest level of education completed?” (0 = no formal education, 1 = elementary school, 2 = middle school, 3 = high school, 4 = university degree, 5 = Ph.D. or similar). A set of data was downloaded from the Prolific website: age, employment status, country of birth, student status, socioeconomic status, sex, nationality, current country of residence, and first language. Go to https://www.prolific.co/ for the exact wording of each question. In particular, subjective socioeconomic status (SES) was measured by the MacArthur Scale of Subjective Social Status (Adler et al., 2008), which asks respondents to choose a number from 1 to 10 representing where they stand in society, with 1 representing the bottom (those who are worst off) and 10 representing the top (those who are best off).

### Statistical Analyses

We implemented a structural equation modeling analysis in Mplus 8 (Muthén and Muthén, 2017) to estimate parameters and test hypotheses concerning the relationships depicted in Figure 1. Ten latent variables were defined according to the coding scheme for composite scores (see “Protective behaviors,” “Risk perception,” “Attitude,” “Experience,” “Cultural worldviews,” and “Normative conducts”). Thus, hierarchy–egalitarianism, individualism–communitarianism, and direct and indirect experience of COVID-19 were the exogenous latent variables; social norms, affective attitude, feelings of risk, risk analysis, promoting hygiene and cleaning, and avoiding social closeness were the endogenous ones. Because we measured all the latent variables by multiple Likert-type (or ordered categorical) items, we carried out the analysis using robust weighted least squares estimators (WLSMV). This method makes no distributional assumptions and is recommended to handle ordinal data (Rhemtulla et al., 2012).

Besides model χ^2^, we assessed the model’s fit using other descriptive indexes: comparative fit index (CFI), Tucker–Lewis index (TLI), root mean square error of approximation (RMSEA), and standardized root mean square residual (SRMR). According to Hu and Bentler (1999), CFI and TLI greater than 0.95 indicate a good fit of the model, with values above 0.90 deemed acceptable. A good fit is also supported by RMSEA and SRMR lower than 0.06 and 0.08, respectively. We relied on four criteria to assess the quality of the measurement model. First, all empirical indicators should load on the corresponding latent variables above 0.50 (*indicator reliability*). Second, the composite reliability (CR) of each latent variable was expected to be greater than 0.60 or better above 0.70 (*construct reliability*). Third, the average variance extracted (AVE), an index of the proportion of variance in the indicators that was accounted for by the corresponding latent variable, should be greater than 0.50 or higher (*convergent validity*). Lastly, the square roots of the AVE for each latent variable should be greater than the estimated correlations of that latent variable with other variables in the model (*discriminant validity*).

The significance of indirect effects was tested using bias-corrected bootstrap confidence intervals with 1,000 resamplings. Each indirect effect represents the average increase in protective behavior accounted for by direct and indirect experience of COVID-19 and hierarchical and individualistic worldviews through specific intermediate variables, like affective attitude, risk perceptions, and social norms. Standardized indirect effects around 0.02, 0.13, and 0.26 represent small, medium, and large effect size thresholds, respectively (Hayes and Rockwood, 2017).

## Results

### Descriptive Analysis

Our descriptive analysis started with examining the average composite scores reported by different sociodemographic groups (Table 3). Younger participants were less afraid of coronavirus and were less apt to avoid social closeness than older ones. Gender was the sociodemographic variable that affected participants’ ratings the most. In particular, men rated the coronavirus as less risky, in terms of both feelings of risk and risk analysis, and they reported fewer negative feelings than women did. Men held a more individualistic and hierarchical worldview than women. Also, men perceived significant others to protect themselves to a lesser extent and felt less socially pressured to reduce coronavirus risk. Concerning protective behaviors, men reported less hygiene and cleaning and avoided social closeness to a lesser extent than women did. More educated and higher SES participants were higher in social norms, communitarianism, and avoiding social closeness. Higher SES participants also promoted more hygiene and cleaning. The more educated groups endorsed a more egalitarian worldview. Consistent with epidemiological data, people living in the southern regions of the country had significantly lesser direct experience of COVID-19 as a cause of death or suffering and perceived a lower probability of infection. However, they also reported more hygiene and cleaning than those living in the northern areas, but not a greater tendency to avoid social closeness.

**TABLE 3:** Descriptive analysis of composite scores by sociodemographic variables.

**Variable**		**Indirect Experience**		**Direct Experience**		**Affective Attitude**		**Feelings of Risk**		**Risk Analysis**		**Social Norms**		**Hierarchy-Egalitarianism**		**Individualism-Communitarianism**		**Promoting hygiene and cleaning**		**Avoiding Social Closeness**	
**Age group**	***n***	***M***	***SD***	***M***	***SD***	***M***	***SD***	***M***	***SD***	***M***	***SD***	***M***	***SD***	***M***	***SD***	***M***	***SD***	***M***	***SD***	***M***	***SD***
	
18–25 years	328	9.09	1.72	0.33	0.69	2.01	0.71	**2.87**	**0.79**	3.22	0.84	6.03	1.02	2.10	1.08	3.16	1.04	4.60	0.93	**5.05**	**0.88**
26–35 years	180	9.03	1.72	0.26	0.59	1.95	0.76	**3.08**	**0.75**	3.17	0.83	6.14	0.81	2.23	1.07	3.10	1.08	4.73	0.84	**5.38**	**0.65**
36–45 years	65	9.31	1.84	0.28	0.63	1.77	0.79	**3.32**	**0.83**	3.23	0.76	6.27	0.82	2.44	1.21	3.11	1.05	4.72	1.04	**5.40**	**0.93**
*F*, η*^2^*		0.620, 2%		0.65 0, 2%		2.86 1, 0%		11.00***, **3.7%**		0.20 0, 1%		2.18 0, 8%		2.82 1, 0%		0.25 0, 1%		1.51 0, 5%		11.58***, 3.9%	
	
**Gender**	***n***	***M***	***SD***	***M***	***SD***	***M***	***SD***	***M***	***SD***	***M***	***SD***	***M***	***SD***	***M***	***SD***	***M***	***SD***	***M***	***SD***	***M***	***SD***
	
Male	308	9.00	1.78	0.31	0.67	**2.13**	**0.76**	**2.79**	**0.76**	**3.12**	**0.82**	**5.95**	**0.95**	**2.50**	**1.12**	**3.31**	**1.08**	**4.50**	**0.94**	**5.07**	**0.88**
Female	264	9.21	1.68	0.29	0.64	**1.77**	**0.66**	**3.21**	**0.76**	**3.31**	**0.83**	**6.26**	**0.90**	**1.80**	**0.94**	**2.93**	**0.99**	**4.83**	**0.86**	**5.34**	**0.75**
*F*, η*^2^*		2.05 0, 4%		0.21 0, 0%		35.27***, 5.8%		44.01***, 7.2%		7.65**, 1.3%		15.67***, 2.7%		63.19***, 10.0%		18.94***, **3.2%**		18.36***, 3.1%		15.15***, 2.6%	
	
**SES group**	***n***	***M***	***SD***	***M***	***SD***	***M***	***SD***	***M***	***SD***	***M***	***SD***	***M***	***SD***	***M***	***SD***	***M***	***SD***	***M***	***SD***	***M***	***SD***
	
Low	55	8.84	1.80	0.16	0.37	2.00	0.80	2.92	0.86	2.97	0.87	**5.78**	**1.12**	2.34	1.09	**3.43**	**1.22**	**4.32**	**1.06**	**4.93**	**1.04**
Middle	311	9.03	1.72	0.31	0.69	1.97	0.73	2.98	0.79	3.20	0.82	**6.09**	**0.93**	2.09	1.04	**3.21**	**1.06**	**4.65**	**0.91**	**5.25**	**0.75**
High	206	9.26	1.74	0.33	0.64	1.94	0.74	3.01	0.77	3.27	0.82	**6.18**	**0.89**	2.27	1.17	**2.96**	**0.96**	**4.74**	**0.87**	**5.18**	**0.87**
*F*, η*^2^*		1.78 0, 6%		1.39 0, 5%		0.26 0, 1%		0.31 0, 1%		2.94 1, 0%		4.00*, **1.4%**		2.51 0, 9%		5.89**, **2.0%**		4.65*, **1.6%**		3.47*, **1.2%**	
	
**Education**	***n***	***M***	***SD***	***M***	***SD***	***M***	***SD***	***M***	***SD***	***M***	***SD***	***M***	***SD***	***M***	***SD***	***M***	***SD***	***M***	***SD***	***M***	***SD***
	
Middle	24	8.83	1.58	0.38	0.71	2.09	0.73	2.89	0.89	3.21	0.71	**5.52**	**1.18**	**2.58**	**1.53**	**3.33**	**1.31**	4.45	0.93	**4.67**	**0.85**
High	291	9.07	1.73	0.27	0.60	1.98	0.73	2.92	0.79	3.18	0.88	**6.04**	**0.96**	**2.26**	**1.08**	**3.22**	**0.99**	4.66	0.94	**5.15**	**0.89**
University	240	9.17	1.74	0.32	0.69	1.93	0.76	3.05	0.79	3.23	0.79	**6.19**	**0.89**	**2.07**	**1.07**	**3.06**	**1.11**	4.66	0.89	**5.27**	**0.75**
Ph.D.	18	8.83	2.09	0.50	0.86	1.89	0.70	3.20	0.74	3.31	0.65	**6.50**	**0.45**	**1.85**	**0.87**	**2.60**	**0.72**	4.80	0.85	**5.56**	**0.60**
*F*, η*^2^*		0.51 0, 3%		0.94 0, 5%		0.48 0, 3%		1.78 0, 9%		0.27 0, 1%		5.42**, **2.8%**		2.98*, **1.5%**		2.88*, **1.5%**		0.57 0, 3%		5.18**, **2.7%**	
	
**Zone**	***n***	***M***	***SD***	***M***	***SD***	***M***	***SD***	***M***	***SD***	***M***	***SD***	***M***	***SD***	***M***	***SD***	***M***	***SD***	***M***	***SD***	***M***	***SD***
	
North	259	9.14	1.69	**0.44**	**0.81**	1.94	0.71	2.98	0.78	**3.31**	**0.85**	6.06	0.99	2.23	1.13	3.13	1.04	**4.55**	**0.92**	5.12	0.82
Center	148	9.09	1.72	**0.26**	**0.52**	1.97	0.80	3.02	0.80	**3.21**	**0.77**	6.12	0.92	2.11	1.11	3.09	1.07	**4.70**	**0.88**	5.23	0.86
South	165	9.02	1.81	**0.12**	**0.37**	2.00	0.73	2.96	0.81	**3.04**	**0.82**	6.11	0.88	2.15	1.03	3.19	1.06	**4.77**	**0.93**	5.28	0.83
*F*, η*^2^*		0.23 0, 1%		12.39***, **4.2%**		0.35 0, 1%		0.18 0, 1%		5.26**, **1.8%**		0.24 0, 1%		0.66 0, 2%		0.33 0, 1%		3.13*, **1.1%**		2.23 0, 8%	
	
	***n***	***M***	***SD***	***M***	***SD***	***M***	***SD***	***M***	***SD***	***M***	***SD***	***M***	***SD***	***M***	***SD***	***M***	***SD***	***M***	***SD***	***M***	***SD***
	
**Total Sample**	572	9.10	1.73	0.30	0.65	1.96	0.74	2.98	0.79	3.21	0.83	6.09	0.94	2.18	1.10	3.14	1.05	4.65	0.91	5.19	0.83

Significant differences are in boldface. *p < 0.05. **p < 0.01. ***p < 0.001.

Table 4 reports intercorrelations among composite scores either controlling or not controlling for sociodemographic variables (above and below the diagonal, respectively). Promoting hygiene and cleaning and avoiding social closeness were both associated with risk perception variables (i.e., affective attitude, feelings of risk, and risk analysis), cultural worldviews (i.e., hierarchy–egalitarianism and individualism–communitarianism), and social norms. Controlling for sociodemographic factors, hierarchy–egalitarianism was no longer associated with the two types of protective behaviors. Experience of COVID-19, both direct and indirect, was only marginally associated with protective behaviors. A greater indirect experience was associated with increased feelings of risk and risk analysis as well as a more negative affective attitude. Individualism was associated with decreased risk perceptions and decreased negative emotions to a larger extent than hierarchy. In turn, greater individualism and hierarchy were associated with less perceived pressure to conform to social norms. Although the previous descriptive analyses highlighted significant differences in the average values of the composite scores, the correlations among the variables do not differ by age, gender, SES, education, and zone.

**TABLE 4:** Intercorrelations of composite scores.

	1	2	3	4	5	6	7	8	9	10
(1) Indirect Experience		0.04	−0.21***	0.21***	0.15***	–0.01	0.02	–0.05	0.07	0.02
(2) Direct Experience	0.05		–0.05	0.08	0.09*	0.06	–0.03	–0.06	0.10	0.05
(3) Affective Attitude	−0.22***	–0.04		−0.57***	−0.28***	−0.16***	0.11**	0.17***	−0.22***	−0.20***
(4) Feelings of Risk	0.22***	0.07	−0.60***		0.46***	0.04	0.03	−0.16***	0.32***	0.21***
(5) Risk Analysis	0.17***	0.12**	−0.30***	0.45***		0.08	−0.09*	−0.21***	0.18***	0.20***
(6) Social Norms	0.01	0.06	−0.19***	0.10*	0.10*		−0.28***	−0.26***	0.11***	0.18***
(7) Hierarchy-Egalitarianism	0.01	–0.02	0.16***	–0.03	−0.11**	−0.30***		0.27***	–0.06	−0.19***
(8) Individualism–Communitarianism	–0.07	–0.07	0.21***	−0.20***	−0.24***	−0.30***	0.30***		–0.06	−0.21***
(9) Promoting Hygiene and Cleaning	0.09*	0.07	−0.25***	0.36***	0.18***	0.15***	−0.10*	−0.10*		0.49***
(10) Avoiding Social Closeness	0.03	0.03	−0.24***	0.27***	0.20***	0.22***	−0.21***	−0.24***	0.51***	

Partial intercorrelations controlling for age, gender, SES, education, and zone are presented above the diagonal, and Pearson intercorrelations are presented below the diagonal. * *p* < 0.05. **p < 0.01. ***p < 0.001.

### Structural Equation Modeling

**FIGURE 2:**
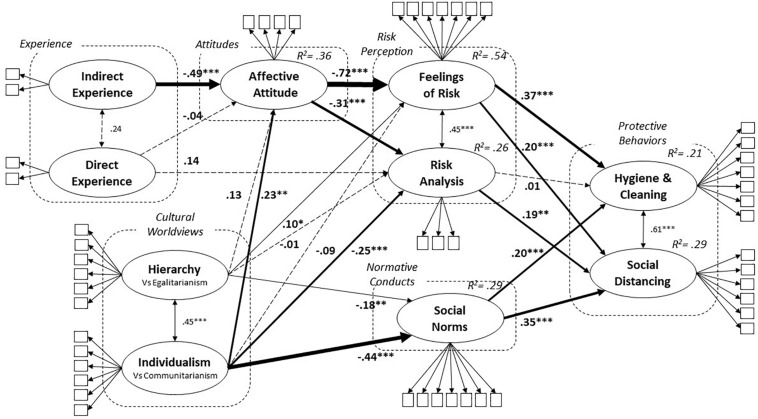
The COVID-19 Risk Perception and Protective Behavior Model with standardized path coefficients for causal paths represented by straight single-headed arrows. Non significant correlations among exogenous variables omitted. Coefficients flagged with asterisks are significantly different from zero, *p < 0.05, **p < 0.01, ***p < 0.001.

Although the model depicted in Figure 2 was significant (χ^2^ = 2712.49; df = 1148; *p* < 0.001), its fit was overall acceptable (CFI = 0.922; TLI = 0.917; RMSEA = 0.049; *p*-close = 0.777; SRMR = 0.066). Regarding the quality of the measurement model, all factor loadings were statistically significant (Table 5). Except for indicators of indirect experience and two indicators of individualism–communitarianism, all items loaded on the corresponding latent variables above 0.50, supporting the indicator reliability in 46 of 50 cases (92%). Table 6 summarizes the model-based CR coefficients and the AVE for each latent variable, and the estimated correlations among all latent variables in the model. Except for indirect experience, the CRs were well above the recommended threshold of 0.60, ranging in most cases from 0.75 to 0.92 for all the latent variables in the model. All constructs but indirect experience were reliably measured.

**TABLE 5:** Latent variables and factor loadings.

*Indirect Experience*					*Feelings of Risk*					*Promoting Hygiene and Cleaning*				
*Indicator*	λ	*SE(λ)*	*t-value*	*p-value*	*Indicator*	λ	*SE(λ)*	*t-value*	*p-value*	*Indicator*	λ	*SE(λ)*	*t-value*	*p-value*
EXPIND1	0.27	0.07	4.14	0.000	RISKAFF1	0.90	0.01	69.54	0.000	PREVBEH1	0.70	0.04	18.79	0.000
EXPIND2	0.50	0.12	4.39	0.000	RISKAFF2	0.92	0.01	90.46	0.000	PREVBEH2	0.69	0.03	21.80	0.000
***Direct Experience***					RISKPERC1	0.83	0.02	50.21	0.000	PREVBEH7	0.71	0.03	21.50	0.000
***Indicator***	**λ**	***SE(λ)***	***t-value***	***p-value***	RISKPERC2	0.74	0.02	32.10	0.000	PREVBEH9	0.62	0.04	17.71	0.000

EXPDIR1	0.91	0.35	2.64	0.008	RISKPERC3	0.80	0.02	41.60	0.000	PREVBEH10	0.74	0.04	20.16	0.000
EXPPDIR2	0.83	0.29	2.86	0.004	RISKEXP1	0.53	0.03	16.08	0.000	PREVBEH12	0.67	0.03	20.86	0.000
***Hierarchy-Egalitarism***					RISKEXP2	0.63	0.03	23.39	0.000	PREVBEH13	0.60	0.04	15.42	0.000
***Indicator***	**λ**	***SE(λ)***	***t-value***	***p-value***	***Risk Analysis***					***Avoiding Social Closeness***				
		
WVH1	0.80	0.02	35.72	0.000	***Indicator***	**λ**	***SE(λ)***	***t-value***	***p-value***	***Indicator***	**λ**	***SE(λ)***	***t-value***	***p-value***
WVH2	0.81	0.02	37.17	0.000	RISKPROB	0.51	0.04	12.77	0.000	PREVBEH3	0.76	0.04	20.59	0.000
WVH3	0.82	0.02	40.09	0.000	RISKCOND1	0.77	0.04	18.87	0.000	PREVBEH4	0.79	0.03	29.87	0.000
WVE1 (R)	0.63	0.03	20.49	0.000	RISKCOND2	0.51	0.04	12.13	0.000	PREVBEH5	0.62	0.04	17.88	0.000
WVE2 (R)	0.80	0.02	33.36	0.000	***Social Norms***					PREVBEH6	0.82	0.03	30.95	0.000
WVE3 (R)	0.68	0.03	23.36	0.000	***Indicator***	**λ**	***SE(λ)***	***t-value***	***p-value***	PREVBEH8	0.63	0.04	15.87	0.000
	
***Individualism-Communitarianism***					NORMP1	0.85	0.02	39.12	0.000	PREVBEH11	0.54	0.05	10.28	0.000
***Indicator***	**λ**	***SE(λ)***	***t-value***	***p-value***	NORMP2	0.81	0.03	32.52	0.000	***Affective Attitude***				
		
WVI1	0.72	0.03	21.26	0.000	NORMP3	0.73	0.03	29.09	0.000	***Indicator***	**λ**	***SE(λ)***	***t-value***	***p-value***
		
WVI2	0.66	0.03	19.53	0.000	NORMP4	0.57	0.04	15.59	0.000	AFFATT1	0.79	0.02	38.11	0.000
WVI3	0.71	0.03	23.47	0.000	NORMD1	0.87	0.02	54.65	0.000	AFFATT2	0.90	0.01	69.21	0.000
WVC1 (R)	0.60	0.04	15.44	0.000	NORMD2	0.85	0.02	49.11	0.000	AFFATT3	0.84	0.02	45.53	0.000
WVC2 (R)	0.42	0.04	10.38	0.000	NORMD3	0.77	0.02	33.14	0.000	AFFATT4	0.88	0.02	60.15	0.000
WVC3 (R)	0.41	0.04	10.09	0.000										

*EXPIND, indirect experience; EXPDIR, direct experience; RISKAFF, affective risk perception; RISKPERC, general risk perception; RISKEXP, experiential risk perception; RISKCOND, conditional risk perception; RISKPROB, perceived likelihood; WVH, hierarchy; WVE, egalitarianism; WVI, individualism; WVC, communitarianism; NORMP, prescriptive norms; NORMD, descriptive norms; PREVBEH, protective behavior; AFFATT, affect.*

**TABLE 6:** Reliability and validity of the latent variables.

	CR	AVE	1	2	3	4	5	6	7	8	9	10
(1) Indirect Experience	0.26	0.16	**0.40**									
(2) Direct Experience	0.86	0.76	0.24***	**0.87**								
(3) Affective Attitude	0.91	0.73	–0.51***	–0.10*	**0.85**							
(4) Feelings of Risk	0.91	0.60	0.39***	0.08	–0.73***	**0.77**						
(5) Risk Analysis	0.66	0.40	0.24***	0.20***	–0.42***	0.58***	**0.63**					
(6) Social Norms	0.92	0.61	0.07	0.05	–0.20***	0.15***	0.20***	**0.78**				
(7) Hierarchy-Egalitarianism	0.89	0.58	0.02	0.01	0.22***	–0.10*	–0.19***	–0.38***	**0.76**			
(8) Individualism–Communitarianism	0.75	0.34	–0.18***	–0.12**	0.37***	–0.31***	–0.39***	–0.52***	0.45***	**0.58**		
(9) Promoting hygiene and cleaning	0.86	0.46	0.16***	0.04	–0.32***	0.41***	0.27***	0.26***	–0.12**	–0.23***	**0.68**	
(10) Avoiding Social Closeness	0.85	0.49	0.15***	0.07	–0.30***	0.36***	0.37***	0.42***	–0.19***	–0.32***	0.68***	**0.70**

Latent correlations among the variables in the model are presented below the diagonal. Squared roots of AVE coefficients in the diagonal are in boldface. AVE, average variance extracted; CR, composite reliability. * *p* < 0.05. **p < 0.01. ***p < 0.001.

As showed by AVEs reported in Table 6, the convergent validity criterion was fully achieved for direct experience, affective attitude, feelings of risk, social norms, individualism–communitarianism, and hierarchy–egalitarianism. Risk analysis, avoiding social closeness, and promoting hygiene and cleaning were close to the recommended standard of convergent validity (i.e., AVE > 0.50), whilst indirect experience failed to meet the psychometric requirement. Importantly, the square roots of AVEs (in the diagonal of Table 6) were higher than the estimated correlations of the latent variables with other latent variables in the model, thus meeting the criterion for discriminant validity. Taken together, these results supported the quality of the measurement model for all latent variables except indirect experience, as well as our decision to consider descriptive and prescriptive social norms as a single latent variable and to include experiential risk indicators in the latent variable of feelings of risk.

Figure 2 shows the estimated structural coefficients and the *R*^2^ for the endogenous variables. The model explained 21% and 29% of the variance in promoting hygiene and cleaning and avoiding social closeness, respectively; 54% and 26% in feelings of risk and risk analysis, respectively; 36% in affective attitude; and 29% in social norms. The most important endogenous variables were promoting hygiene and cleaning and avoiding social closeness. Feelings of risk and social norms (in decreasing importance) were significantly associated with both types of protective behaviors, whilst risk analysis was significant only with avoiding social closeness (Figure 2). Thus, people feeling more afraid of coronavirus not only implemented more proactive behaviors, like cleaning hands, sanitizing surfaces, and wearing masks, but also were more apt to avoid social closeness, refraining from exchanging gestures of affection or greetings such as hugs and handshakes. In contrast, a higher perceived likelihood of infection motivated one’s avoidance of social closeness.

Before presenting the results concerning the antecedents of feelings of risk and risk analysis, it is worth describing the relationships of social norms with both kinds of protective behaviors. In particular, people who thought that significant others were performing appropriate behaviors to protect themselves and felt socially pressured to comply were also more likely to avoid social closeness and promoting hygiene and cleaning. Social norms were supposed to be influenced by cultural worldviews. This hypothesis was confirmed by the significant structural coefficients of hierarchy–egalitarianism and individualism–communitarianism (see Figure 2). Notably, the latter predictor had a larger effect size, suggesting that holding an individualistic worldview more than a hierarchical one could lead people to believe that significant others protected themselves less and to perceive less social pressure to adhere to protective behaviors.

Affective reactions associated with coronavirus had a central role in the model. First, affective attitude was the best predictor of participants’ feelings of risk and the largest structural coefficient in the model. Second, affective attitude also informed participants’ risk analysis, increasing the perceived probability of being exposed to the risk of infection. According to the theoretical framework depicted in Figure 1, we hypothesized that cultural worldviews could shape one’s feelings of risk, risk analysis, and affective attitude. These hypotheses were only partially supported. Although hierarchy–egalitarianism and individualism–communitarianism were statistically significantly related to feelings of risk, the structural coefficients were small. Only individualism–communitarianism was among the significant predictors of risk analysis, with an effect size about as large as that assessed for affective attitude. In particular, individuals oriented toward a more individualistic worldview perceived a lower probability of becoming infected than those with a more communal worldview. Both hierarchy–egalitarianism and individualism–communitarianism were associated with a less negative affective attitude toward coronavirus, but only the latter attained the conventional levels of statistical significance. As expected, more indirect experience of COVID-19 was the strongest predictor of negative affective attitude. Taken together, experience and cultural worldview variables accounted for 36% of the variance in affective attitude.

A subsequent analysis added age, gender, education, socioeconomic status, and zone of the country where participants lived at the time of data collection as exogenous variables, each of which had a direct path to protective behaviors, risk perceptions, affective attitudes, and social norms. The purpose was to increase the variance in endogenous variables accounted for by the model and assess whether the structural coefficients (reported in Figure 2) were robust to differences in sociodemographic factors. Overall, the model controlling for sociodemographic factors accounted for a larger proportion of variance compared to previous analysis: 23% and 37% in promoting hygiene and cleaning and avoiding social closeness, respectively; 56% and 37% in feelings of risk and risk analysis, respectively; 39% in affective attitude; and 30% in social norms. Although all fit indices still were acceptable, and in some cases good (CFI = 0.908; TLI = 0.900; RMSEA = 0.047; *p*-close = 0.988; SRMR = 0.076), the model was statistically significant (χ^2^ = 3085.43; df = 1368; *p* = 0.000), and the TLI, which penalizes models that estimate many parameters, was barely sufficient. The inspection of structural coefficients for sociodemographic factors revealed the following statistically significant effects (all *p*-s < 0.05): SES and zone on promoting hygiene and cleaning; age and zone on avoiding social closeness; age and gender on feelings of risk, affective attitude, and social norms; zone on risk analysis; and SES on social norms. The direction and interpretation of these effects reflected previous descriptive analyses reported in Table 3. Notably, the new analysis did not alter the significance of structural coefficients (previously reported in Figure 2) except for the link between feelings of risk and avoiding social closeness, which was no longer significant controlling for demographics (β = −0.03; *p* = 0.773). Another ostensible difference between analyses was the change in the link between risk analysis and avoiding social closeness, which doubled its effect size by controlling for sociodemographic variables (β = 0.44; *p* = 0.000). These changes were due to the associations of age and gender with the latent variables in the model, as showed by a control analysis in which only removing both variables from the model restored the significance of the relationship between feelings of risk and avoiding social closeness (β = 0.15; *p* = 0.019) and lessened the effect size of risk analysis.

Bootstrap tests of indirect effects are reported in Table 7. Participants’ indirect experience of COVID-19 was significantly associated with undertaking more promoting hygiene and cleaning through affective attitudes and feelings of risk. The indirect effect of positive affective attitude on promoting hygiene and cleaning through decreasing one’s feelings of risk was also statistically significant, with a large effect size. This result indicated that affective risk perception mediated the relationship between one’s affective attitude toward coronavirus and a specific type of protective behavior involving hand-cleaning, surface disinfection, and wearing facemasks. The analysis of indirect effects showed that social norms had a role as a mediating variable in the relationship between worldviews and adherence to protective measures. In particular, a more communitarian (and less individualistic) worldview led to greater adherence to avoiding social closeness and promoting hygiene and cleaning through the increased perception that significant others would behave in such a way and would approve.

**TABLE 7:** Bootstrap tests of indirect effects.

			Promoting Hygiene and Cleaning				Avoiding Social Closeness			
			Adjusted Estimates (controlling for sociodemographic variables)							
*Indirect Effect*			*Est.*	*95% CI*	*99% CI*	*St. Est.*	*Est.*	*95% CI*	*99% CI*	*St. Est.*
Ind.Exp. >	Aff.Att. >	Feel.Risk >	**0.35**	**[0.13; 0.76]**	**[0.00; 0.76]**	0.10	–0.03	[–0.90; 0.30]	[–1.76; 0.43]	–0.01
Ind.Exp. >	Aff.Att. >	Risk.Analysis >	0.03	[–0.08; 0.33]	[–0.08; 0.63]	0.01	**0.31**	**[0.04; 1.44]**	**[0.03; 2.29]**	0.08
Dir.Exp. >	Aff.Att. >	Feel.Risk >	0.00	[–0.06; 0.03]	[–0.06; 0.03]	0.00	0.00	[–0.05; 0.03]	[–0.07; 0.09]	0.00
Dir.Exp. >	Aff.Att. >	Risk.Analysis >	0.00	[–0.02; 0.03]	[–0.02; 0.03]	0.00	0.00	[–0.13; 0.06]	[–0.13; 0.08]	0.00
	Aff.Att. >	Feel.Risk >	**–0.17**	**[–0.33; –0.07]**	[–0.33; 0.00]	–0.23	0.02	[–0.14; 0.39]	[–0.14; 0.54]	0.02
	Aff.Att. >	Risk.Analysis >	–0.02	[–0.17; 0.05]	[–0.38; 0.05]	–0.02	–**0.15**	**[**–**0.63;** –**0.03]**	**[**–**1.45;** –**0.02]**	–0.17
Hier./Ega. >	Aff.Att. >	Feel.Risk >	–0.02	[–0.08; 0.00]	[–0.08; 0.02]	–0.02	0.00	[–0.03; 0.11]	[–0.06; 0.14]	0.00
Hier./Ega. >	Aff.Att. >	Risk.Analysis >	0.00	[–0.03; 0.01]	[–0.03; 0.02]	0.00	–0.01	[–0.16; 0.01]	[–0.16; 0.07]	–0.02
Ind./Com. >	Aff.Att. >	Feel.Risk >	–**0.05**	**[**–**0.19;** –**0.01]**	[–0.19; 0.09]	–0.05	0.01	[–0.09; 0.15]	[–0.09; 0.27]	0.00
Ind./Com. >	Aff.Att. >	Risk.Analysis >	–0.01	[–0.07; 0.02]	[–0.36; 0.03]	0.00	–0.05	[–0.29; 0.00]	[–0.62; 0.00]	–0.04
	Hier./Ega. >	Feel.Risk >	0.04	[0.01; 0.08]	[–0.02; 0.08]	0.05	0.00	[–0.11; 0.04]	[–0.30; 0.04]	0.00
	Hier./Ega. >	Risk.Analysis >	0.00	[–0.06; 0.01]	[–0.07; 0.02]	0.00	–0.03	[–0.14; 0.02]	[–0.20; 0.03]	–0.03
	Ind./Com. >	Feel.Risk >	–0.03	[–0.09; 0.01]	[–0.09; 0.03]	–0.02	0.00	[–0.04; 0.18]	[–0.08; 0.22]	0.00
	Ind./Com. >	Risk.Analysis >	–0.01	[–0.11; 0.05]	[–0.34; 0.06]	–0.01	–0.13	[–0.38; 0.07]	[–0.57; 0.10]	–0.10
	Hier./Ega. >	Soc.Norms >	–0.02	[–0.09; 0.00]	[–0.09; 0.01]	–0.03	–**0.04**	**[**–**0.09;** –**0.01]**	[–0.09; 0.00]	–0.04
	Ind./Com. >	Soc.Norms >	–**0.07**	**[**–**0.17;** –**0.02]**	**[**–**0.17;** –**0.02]**	–0.06	–**0.12**	**[**–**0.32;** –**0.04]**	**[**–**0.37;** –**0.02]**	–0.10

			**Unadjusted Estimates**							

Ind.Exp. >	Aff.Att. >	Feel.Risk >	**0.44**	**[0.99; 0.14]**	[–0.07; 0.99]	0.13	0.28	[0.93; –0.30]	[–0.66; 0.93]	0.07
Ind.Exp. >	Aff.Att. >	Risk.Analysis >	0.01	[0.33; –0.09]	[–0.09; 0.40]	0.00	0.12	[0.86; –0.04]	[–0.04; 1.28]	0.03
Dir.Exp. >	Aff.Att. >	Feel.Risk >	–0.01	[0.01; –0.10]	[–0.10; 0.02]	–0.01	0.00	[0.01; –0.12]	[–0.12; 0.01]	–0.01
Dir.Exp. >	Aff.Att. >	Risk.Analysis >	0.00	[0.00; –0.03]	[–0.04; 0.01]	0.00	0.00	[0.01; –0.05]	[–0.05; 0.01]	0.00
	Aff.Att. >	Feel.Risk >	–**0.20**	**[**–**0.08;** –**0.33]**	[–0.33; 0.01]	–0.26	–0.13	[0.14; –0.24]	[–0.24; 0.40]	–0.14
	Aff.Att. >	Risk.Analysis >	0.00	[0.03; –0.18]	[–0.25; 0.04]	–0.01	–0.06	[0.01; –0.46]	[–0.84; 0.01]	–0.06
Hier./Ega. >	Aff.Att. >	Feel.Risk >	–0.03	[0.00; –0.09]	[–0.09; 0.01]	–0.03	–0.02	[0.01; –0.13]	[–0.13; 0.03]	–0.02
Hier./Ega. >	Aff.Att. >	Risk.Analysis >	0.00	[0.00; –0.05]	[–0.05; 0.02]	0.00	–0.01	[0.00; –0.16]	[–0.16; 0.04]	–0.01
Ind./Com. >	Aff.Att. >	Feel.Risk >	–0.06	[0.00; –0.19]	[–0.22; 0.02]	–0.06	–0.04	[0.05; –0.25]	[–0.25; 0.15]	–0.03
Ind./Com. >	Aff.Att. >	Risk.Analysis >	0.00	[0.02; –0.08]	[–0.18; 0.03]	0.00	–0.02	[0.00; –0.25]	[–0.69; 0.01]	–0.01
	Hier./Ega. >	Feel.Risk >	**0.03**	**[0.06; 0.00]**	[–0.02; 0.08]	0.04	0.02	[0.05; –0.03]	[–0.06; 0.07]	0.02
	Hier./Ega. >	Risk.Analysis >	0.00	[0.01; –0.04]	[–0.08; 0.04]	0.00	0.00	[0.03; –0.12]	[–0.16; 0.15]	0.00
	Ind./Com. >	Feel.Risk >	–0.03	[0.01; –0.11]	[–0.11; 0.03]	–0.03	–0.02	[0.02; –0.10]	[–0.10; 0.07]	–0.02
	Ind./Com. >	Risk.Analysis >	0.00	[0.05; –0.10]	[–0.15; 0.07]	0.00	–0.06	[0.01; –0.32]	[–0.63; 0.14]	–0.05
	Hier./Ega. >	Soc.Norms >	–0.03	[0.00; –0.08]	[–0.08; 0.02]	–0.04	–0.06	[0.00; –0.15]	[–0.15; 0.03]	–0.06
	Ind./Com. >	Soc.Norms >	–**0.09**	**[**–**0.04;** –**0.21]**	**[**–**0.24;** –**0.02]**	–0.09	–**0.19**	**[**–**0.07;** –**0.43]**	**[**–**0.51;** –**0.07]**	–0.15

*Significant estimates are in boldface. Est., Point Estimate; St. Est, Standardized Estimate; Ind.Exp., Indirect experience; Dir.Exp, Direct experience; Aff.Att., Affective Attitude; Feel. Risk, Feelings of risk; Risk.Analysis, Risk analysis; Hier./Ega., Hierarchy-Egalitarianism; Ind./Com., Individualism–Communitarianism.*

All indirect effects mentioned above remained statistically significant when controlling for sociodemographic factors. However, the adjusted estimates revealed that participants’ indirect experience of COVID-19 was significantly associated with avoiding social closeness through affective attitudes and risk analysis. Moreover, the indirect effect of affective attitude on avoiding social closeness through decreasing one’s risk analysis was also statistically significant, with a medium effect size. This result indicated that a more deliberate risk judgment (i.e., perceived likelihood) mediated the relationship between one’s affective attitude toward coronavirus and inhibition of habitual social behaviors, such as hugs and handshakes with acquaintances. This effect was “masked” by gender and age differences, which suppressed the link between risk analysis and avoiding social distancing. In our model, individualism (vs. communitarianism) predicted the affective attitude. However, the indirect effect of individualism on promoting hygiene and cleaning through affective attitude and feeling of risk was marginally significant (*p* < 0.10) based on unadjusted estimates. Controlling for sociodemographic factors, this effect attained the conventional levels of statistical significance (*p* < 0.05).

## Discussion

The present study offers insights into people’s adherence to self-reported protective behaviors during the rise of the COVID-19 epidemic in Italy, taking into account the interplay of risk perceptions, social norms, and cultural worldviews. For this purpose, we tested a theoretical model, in which affective and deliberate risk perceptions and social norms were the most proximal predictors of two categories of protective behaviors: promoting hygiene and cleaning and avoiding social closeness. We identified these categories using exploratory factor analysis and confirmed the reliability and validity of the corresponding latent variables in structural equation modeling. Coronavirus is an “invisible” threat against which one method of protection is increasing hygiene. However, because it infects people gathering in social situations, the “invisible” threat materializes in “relationships with others” who become the object of fear. Thus, another way to protect oneself and society is to avoid social closeness. The two categories of protective behaviors tapped into the twofold way in which the spread of infection can be fought and controlled: cutting out the “invisible” and “relational” risks. The model also included more distal predictors, like affective attitude, experience of COVID-19, and cultural worldviews, whose indirect relations with protective behaviors were also evaluated.

The hypothesized model fitted to a national sample was an acceptable fit and provided evidence for two major pathways through which Italian citizens have engaged in protective behaviors. The first pathway has led to increasing compliance with promoting hygiene and cleaning and was triggered by an affective evaluation of coronavirus and mediated by an affective appraisal of risk. The second pathway involved cultural worldviews as predictors and social norms as mediators and was as important for avoiding social closeness as for promoting hygiene and cleaning. The two pathways accounted for a fair amount of variance in health-protective actions. Many earlier studies have also shown a positive association between risk perceptions and health behavior, but the effect sizes were small, ranging from 0.01 to 0.20, with a mean of around 0.14 (Harrison et al., 1992; Floyd et al., 2000). Because of such scarce predictive power, some researchers have maintained that risk perceptions are of limited importance for predicting behaviors, especially those pertaining to the use of protective measures and personal protective equipment (Rundmo, 1996, 2000; Christian et al., 2009; Leiter et al., 2009). But others argued that most of these studies equated risk perceptions with the perceived likelihood of harm (Brewer et al., 2007). Our study adds to this debate, suggesting that research has to consider the affective component in risk perception other than the perceived likelihood, but also calls for including social factors, like worldviews and social norms, to enhance the prediction of behavior.

### Risk Perceptions as an Antecedent of Protective Behavior

Modern theories of risk perception maintain that people perceive hazards prevalently through an affective/experiential way and a deliberate/analytical one (Slovic et al., 2004). In keeping with this view, we included in the model two latent variables — risk analysis and feelings of risk — which achieved high reliability and validity standards. In line with predictions, risk-as-feelings was the best predictor of protective behaviors involving hand-cleaning, surface disinfection, and wearing facemasks. The relation of feelings of risk with social distancing behaviors was instead weak and depended on the effect of sociodemographic factors, a finding that we will discuss in a separate section (see section “Effect of sociodemographic variables”). The reliance on risk-as-feelings in promoting hygiene and cleaning is consistent with previous research and theories highlighting the role of affect experienced at the moment of decision making in predicting protection motivation against a variety of health hazards (Hay et al., 2006; Janssen et al., 2014; Ferrer et al., 2018).

Some authors argued that it is worth distinguishing between experiential and affective components of feelings of risk (Ferrer et al., 2016; Kaufman et al., 2020). Previous research has shown that experiential risk perception, the “gut” feeling of being vulnerable to risk, was associated with performing protective behaviors, such as influenza vaccination and sun protection (Weinstein et al., 2007; Janssen et al., 2011; Ferrer et al., 2016). Although we used some standard questions to assess experiential risk perception of coronavirus, these measures loaded on the same latent variable as the affective indicators and were functionally equivalent in the prediction of protective behaviors. Therefore, perceptions of being vulnerable to coronavirus triggered hygiene and cleaning behaviors to the same extent as being worried about coronavirus, a feeling that reflects the affective component. Although there are many ways to measure experiential risk, our results are in agreement with Slovic et al. (2004), who maintain the affective and experiential components of risk perceptions part of individuals’ intuitive and instinctive reactions to hazards.

The more deliberative and analytical evaluation of risk perception — that is, the perceived likelihood of being infected — impacted only social distancing. While the finding that the perceived probability of infection failed to predict hygiene and cleaning behaviors was in keeping with the literature emphasizing the primacy of affective processes over deliberative ones (Loewenstein et al., 2001; Peters et al., 2006b), the finding that perceived likelihood predicted avoiding social closeness deserves attention. A possible interpretation calls into question the specificity of protective behaviors. Promoting hygiene and cleaning is a non-specific and generalized strategy to cope with an “invisible” threat, for which it is difficult to quantify the likelihood. Instead, social distancing is more crucially related to how much one perceives others to be potential carriers, a piece of information that was delivered communicating the epidemiological statistics in the daily news. Thus, if the perceived probability of others around us being infected was perceived to be high, more social distancing was deemed necessary by the individual to reduce the risk.

### Affective Attitude as an Antecedent of Risk Perception

In keeping with the affect heuristic (Finucane et al., 2000a; Slovic et al., 2004), the holistic affective attitude associated with coronavirus was used by our participants to make risk judgments, not only increasing one’s feelings of risk (with a very large effect size) but also informing a more deliberate risk analysis pertaining the perceived likelihood. In an emotionally salient context (when the risk of contagion was steeply increasing), participants’ affective attitude guided both their affective risk perceptions and their perceived likelihood judgments, although to a lesser extent. This pattern is further confirmation of the central role that affect has in decision making under risk, as repeatedly affirmed in previous research (Loewenstein et al., 2001; Slovic et al., 2004).

Moreover, our model showed that risk perceptions mediated the relations of affective attitude with both types of protective actions (although the indirect effect on avoiding social closeness through risk analysis was significant only when controlling for sociodemographic factors; see “Effect of sociodemographic variables”). We believe this finding is very important because it showed that a negative (or less positive) attitude toward coronavirus could be necessary but not sufficient to activate a protective behavior. It is worth noting that while the affective attitude captured the generalized affective valence associated with the coronavirus, our affective risk perception measure also captured specific emotions and feelings such as fear and worry. This result is consistent with other studies (Lemer et al., 2001; Turner, 2007; Dorison et al., 2020), in which specific negative emotions were better predictors of risk attitudes than negative emotions in general.

Affect induces an automatic action tendency of approach or avoidance: If I like something, I approach it; if I dislike something, I avoid it (Finucane et al., 2000a). However, our study showed that the affective attitude did not directly motivate an active protective behavior. It seems likely that negative affect needs to be further processed to prompt the necessary motivating cognitive resources needed to take action (e.g., disinfect) and self-control automatic responses (e.g., avoid hugs with relatives). This makes the difference between merely avoiding a threat and actively protecting from it. According to our model, affective risk perception represents the link between the affective attitude and behavior. Thus, the affective risk perception does not overlap completely with the affective attitude (as also supported by the discriminant validity of the corresponding latent variables in the model), but it is an autonomous construct, justified by the purpose to motivate human behavior in protective actions requiring commitment.

### Experience and Protective Behavior

How is the affective attitude toward coronavirus shaped? To answer this question, we hypothesized that greater experience of COVID-19 as a cause of death or suffering could be associated with a more negative attitude. Experience with a hazard is important for building an emotional valence that guides subsequent actions and decisions. This process is the core element of motivational salience, the force that drives choices through somatic markers signaling if something is good (or bad) (Bechara and Damasio, 2005; Slovic et al., 2007). In keeping with this view, our study confirmed that the indirect experience of COVID-19 had a significant role in building one’s affective attitude. In particular, having more frequently heard about coronavirus as a cause of death or suffering via the media (newspapers, magazines, radio, television, Internet, etc.) induced greater negative emotions.

Moreover, as shown by the analysis of indirect effects, the indirect experience of COVID-19 was also the most distal predictor of promoting hygiene & cleaning, through increased negative affective attitude and feelings of risk. This finding suggests that a large coverage of deaths and suffering people in the media at the beginning of the epidemic could have changed the emotional attitude toward the coronavirus in a negative sense, triggering feelings of concern and fear, such as to increase compliance with specific behaviors of protection from a new, still unknown, threat. It is worth reminding that at the beginning of the pandemic in Italy a lockdown was issued, and the count of the dead and infected was about 1,300 and 15,000, respectively (whilst, at the moment we are writing, there have been 35,000 deaths and 289,000 confirmed cases across the country). Therefore, one’s affective attitude was almost exclusively shaped by indirect experience through the media.

Relatively few individuals had personal knowledge of people infected by the virus who died or suffered (i.e., 78% of the sample scored 0 on this variable), and only one claimed to have been infected. The finding that the direct experience of COVID-19 had no significant effect on affective attitude or on the perceived probability of getting infected might again reflect the specific timing of the survey. Perhaps, a follow-up study could have a greater chance to detect and assess the role of direct experience in shaping the affective attitudes.

### Cultural Worldviews, Social Norms, and Protective Behaviors

Cultural worldviews can influence risk beliefs and the associated protective behaviors, especially if a government decree has prescribed such behaviors to be adhered to. For instance, previous research has shown that political conservatism or religious fundamentalism led to more polarized attitudes toward risk for controversial science issues (Kahan et al., 2012; Drummond and Fischhoff, 2017). Another study showed that cultural worldviews predicted the likelihood of participating in an institutional green energy program (Cherry et al., 2019). This literature has inspired us to include hierarchic–egalitarian and individualistic–communitarian worldviews in the model as predictors of affective attitude and risk perceptions.

Taking an individualistic stance (compared to having a more communitarian approach) was associated with a less negative affective attitude and a diminished perception of the likelihood of infection, but not with less affective risk perception. By contrast, endorsing a hierarchical (or less egalitarian) worldview did not predict any of these variables. Unbeknown to us when we conducted the study, a recent international survey (Dryhurst et al., 2020) showed that individualism and prosociality (a variable akin to a communitarian worldview) were the best predictors of individual differences in COVID-19 holistic risk perceptions, in the same direction as individualism (vs. communitarianism) did in our model. However, that study used a global index of risk perception, thus entangling the cognitive (likelihood) and affective (worry) dimensions. Deconstructing the risk perception into more specific components, our study added to the literature, showing that individualism (vs. communitarianism) did not predict the affective risk perception when controlling for the antecedent affective attitude. From a cognitive processing perspective, this finding suggests that people who do not expect the society to be committed to fostering collective welfare evaluated the coronavirus less negatively, defusing the subsequent worries. On the contrary, those who thought that the government should do more to advance society’s goals, even if that means limiting the freedom and choices of individuals (communitarian) held a more negative view of coronavirus, were more worried and protected themselves more. The statistical significance of the corresponding indirect effect corroborated this interpretation. Coherently with previous literature (Peters and Slovic, 1996; Kahan et al., 2012; Dryhurst et al., 2020), cultural worldviews had an active and important role in the social construction of risk. According to these theories, worldviews guide our choices and behaviors through our need to be part of a group with which we share important values that are core constructs of our identity (Kahan et al., 2012; Van Boven et al., 2019).

Individualism accounted for protective behaviors also through another pathway that involved social norms. According to the Theory of Normative Social Behavior, prescriptive norms can moderate the influence of descriptive norms on behavior (Rimal and Real, 2005) — i.e., people are more likely to conform to what most others do when they think that significant others expect that behavior from them. In our study, we could not find such an effect because of the high correlation between the measures of the descriptive and prescriptive norms. Separate composite scores were highly inter-correlated, and all empirical indicators of the two norms loaded on a single latent variable with high convergent and discriminant validity in the analysis of structural equations. This result suggests that in the face of public health protection behaviors prescribed by a government authority, the distinction between descriptive and prescriptive norms is more nuanced. Notwithstanding this, social norms not only mediated the effect of individualism but were also the single best predictor of avoiding social closeness and the second-best predictor of promoting hygiene and cleaning. Social norms have been recently included among the social factors that might trigger protective behaviors during the COVID-19 epidemic (Andrews et al., 2020; Bavel et al., 2020). To the best of our knowledge, this is the first study in which social norms were associated with increased compliance with avoiding social closeness and promoting hygiene and cleaning during the COVID-19 epidemic.

### Effect of Sociodemographic Variables

Our descriptive analysis revealed several statistically significant effects of sociodemographic factors on the study variables, the most relevant ones involving gender, age, and zone of the country where one lived during the survey. Notably, women were higher than men on all the components of risk perception, assessing the coronavirus more negatively, feeling more worried and threatened, and perceiving a greater likelihood of infection. This finding is consistent with the well-known “white male” effect, for which males have a relatively low perception of risks compared to women (Finucane et al., 2000b; Chauvin, 2018). In keeping with previous research, women were also more egalitarian and communitarian than men in our study. Moreover, women reported more health protection behavior than men, a finding documented in previous studies of airborne infectious diseases (e.g., Cowling et al., 2010). Younger participants were less worried than older participants about getting infected with coronavirus and less likely to avoid social closeness. These findings probably reflected the fact that it was widely believed that the virus could only kill the elderly with concomitant chronic diseases. Lastly, people living in the south of the country appraised the perceived likelihood of infection as lower than those living in the north, closely reflecting the prevalence rate of the COVID-19 disease.

These findings led us to control for sociodemographic factors in data analysis. Even if the model increased the percentage of variance explained in all endogenous variables, the fit indexes that are more penalized by the complexity of the model showed a clear worsening. Taken together, the structural parameters did not change after controlling for sociodemographic factors, with some notable exceptions regarding the relationships of affective risk perception and perceived likelihood with social distancing. In particular, the adjusted estimates revealed that worrying about coronavirus was no longer associated with social distancing, while the relationship between this behavior and the perceived likelihood of becoming infected increased considerably. The new analysis corroborated the conclusion that keeping social distancing depended more on a deliberate assessment of risk, being crucially related to how much one perceives others to be potential carriers of the virus. Because age and gender were the variables that altered the relationships mentioned above, we interpreted the confounding effect of demographics assuming that being older and women increased worries and feelings of vulnerability as well as increased social distancing behaviors.

### Limitations

One major study limitation is that our dependent measures are self-reported protective behaviors. What people say about their behavior may be different from what they do (or did). Moreover, self-reported protective actions could be inaccurate because of response-set biases (e.g., acquiescence), social desirability, or inaccurate memory. However, self-report is a standard source of information in studies measuring health-protective behaviors in airborne infectious diseases and the COVID-19 outbreak (Cowling et al., 2010; Dryhurst et al., 2020; Hagger et al., 2020; Lin et al., 2020). Future studies might include social desirability scales to check for under- or over-reporting as well as attention checks to improve the validity of self-report data.

Another noteworthy limitation is that the model does not include many variables that could be important to account for individual differences in health behavior. Among these, self-efficacy could be worth inclusion. Self-efficacy refers to an individual’s belief in one’s capabilities to organize and execute the actions required to accomplish something (Bandura, 1977). Self-efficacy has been shown to moderate the impact of risk perceptions on behavior (Rimal et al., 2009). For example, the risk perception attitude (RPA) framework (Rimal and Real, 2003a) suggests that perceptions of risk are the principal motivators for behavior, but efficacy can moderate this effect so that individuals will act to protect against a risk only when both risk perception and self-efficacy are high (Rimal et al., 2009).

We did not assess the “objective” knowledge that people had about COVID-19. Some authors suggest that knowledge could predict reduced risk perception (Siegrist and Árvai, 2020). For example, people with little knowledge of the causes of floods had lower perceptions of flood risk (Botzen et al., 2009). Lower knowledge groups rely on more dimensions of information and less on categorical gist than higher knowledge groups, a tendency described in the Fuzzy-Tracy Theory (Reyna and Lloyd, 2006; Reyna, 2008). Notably, we carried out the study at the beginning of the pandemic in Italy, only two and a half months after Chinese health authorities identified COVID-19 for the first time (i.e., 31 December 2019). Therefore, the coronavirus knowledge was low even among the experts. Future studies might benefit by measuring individual differences in “objective” knowledge using both COVID-19-specific questions or, more broadly, health literacy questions.

Lastly, cognitive factors (e.g., numeracy skills, and cognitive abilities) could influence both risk perception and protective behavior (e.g., Petrova et al., 2017; Cokely et al., 2018). Although we assessed and controlled for participants’ educational level and socioeconomic status (two variables associated with numeracy and cognitive abilities), these sociodemographic factors did not greatly affect our findings. It is possible that more fine-grained assessments of cognitive factors might have a greater impact on risk perception and behaviors.

## Conclusion

Notwithstanding its limitations, the present study provided insights into how experience, affective attitudes, risk perceptions, cultural worldviews, and social norms accounted for individual differences in health-protective behaviors during the first period of the COVID-19 outbreak in Italy. In this context, our findings confirmed the empirical distinction between affective and analytical risk perceptions, underscoring important differences in promoting hygiene and cleaning and avoiding social closeness. Our findings supported the validity of the affect heuristic hypothesis: holding a negative affective attitude toward coronavirus is necessary, although not enough, to shape risk perceptions and the later adoption of protective behaviors. Lastly, we showed social norms as predictors of health behaviors and as a plausible account for why individualistic people were likely to follow the prescribed health-protective behaviors.

### Practical Implications

Because we showed that people distinguished between personal hygiene and social distancing, and different predictors and underlying processes influenced the two categories of protective actions, implications for institutional communication follows.

First, increasing the fear of coronavirus is likely to lead to increasing proactive behaviors based on maintaining hygiene and cleanliness. A communication strategy focused on individualized risk (e.g., reporting empathic stories of single victims who have died or survived with serious consequences) and dreadful images (e.g., intensive care units struggling through coronavirus outbreak or a military fleet carrying coffins of victims) could increase the frequency of washing hands, sanitizing surfaces, and wearing face masks. Institutional communications oriented toward minimizing the death toll, equating COVID-19 to a mere “seasonal flu,” and emphasizing the growing proportion of asymptomatic and young cases might, on the contrary, decrease the behaviors mentioned above. Depending on the specific institutional goals that policymakers intend to achieve, they could use the two different communication strategies.

Our study suggests that increasing the perceived likelihood of contracting the virus may be of little relevance to increasing people’s adherence to hygiene and cleanliness. However, the perceived likelihood of infection was crucial to social distancing. Therefore, it is worth providing people with exact information on the spread of the virus after the peak of the pandemic, when the emotional salience is decreasing. This might help to convince people to keep social distancing when the emergency phase is over and policymakers should prevent a second wave. For instance, this would be helpful in countries in which the lockdown is over (e.g., Italy) and people must return to everyday activities, or in countries in which social distancing is the best strategy to control the pandemic.

Another implication descends from the impact of social norms on protective behaviors. Social norms could be part of communication interventions aimed to promote hygiene and cleaning and social distancing. Looking at what others do (descriptive norms) is an automatic and instinctive way of regulating our behavior. For instance, if our friends keep a safe distance, we conform to their behavior regardless of our risk perceptions. The opposite is also true: if our friends do not refrain from getting close, we do the same. Risk communication should use our natural tendency to follow social norms as a nudging technique (Bicchieri and Dimant, 2019). Broadcasting images of citizens keeping social distance and refraining from exchanging greetings serves as social norms nudges that go toward increasing protective behaviors. However, the media often emphasized examples of the transgression of these safety practices (e.g., showing groups of friends dancing or drinking without social distancing). Although this communication strategy is ubiquitously appealing to the public, it conveys the veiled message that undertaking such protective behaviors is superfluous because people shown in the news do not do it.

## Data Availability Statement

The datasets presented in this study can be found online at: https://osf.io/rq84g/.

## Ethics Statement

The studies involving human participants were reviewed and approved by Ethics Committee for experimentation with the human being of the University of Trento (Protocol n. 2020-020). Written informed consent for participation was not required for this study in accordance with the national legislation and the institutional requirements.

## Author Contributions

LS and ML designed the research, interpreted the results, and wrote the manuscript. LS performed the research. ML analyzed the data. Both authors contributed to the article and approved the submitted version.

## Conflict of Interest

The authors declare that the research was conducted in the absence of any commercial or financial relationships that could be construed as a potential conflict of interest.

## Footnotes

1 http://www.salute.gov.it/portale/news/p3_2_1_1_1.jsp?lingua=italiano&menu=notizie&p=dalministero&id=4156 retrieved on 11.05.2020
