# Supplementary material for: Risk Perception and Protective Behaviors During the Rise of the COVID-19 Outbreak in Italy
Source: Front Psychol. 2021 Jan 13;11:577331. doi: 10.3389/fpsyg.2020.577331 (PMC7838090; doi:10.3389/fpsyg.2020.577331)
Supplement: Supplementary file 1 [file Table_1.DOCX]

Supplementary Materials

**Risk Perception and Protective Behavior During the Rise of the COVID-19 Epidemic in Italy**

Lucia Savadori^1^ & Marco Lauriola^2^

^1^Dipartimento di Economia e Management, Università di Trento, Trento (Italy)

^2^Dipartimento di Psicologia dei Processi di Sviluppo e Socializzazione, Sapienza Università degli Studi di Roma, Roma (Italy)

Correspondence to: Lucia Savadori, lucia.savadori@unitn.it

**Table of Contents**

**Principal Component Analysis of Protective Behaviors (S1-S3)**

S1. Matrix of polychoric correlations

S2. Parallel Analysis and Scree Plots

S3. Promax-rotated factor matrix

**Principal Component Analysis of Risk Perceptions (S4-S6)**

S4. Matrix of polychoric correlations

S5. Parallel Analysis and Scree Plots

S6. Promax-rotated factor matrix

**Principal Component Analysis of Risk Perceptions (S7-S9)**

S7. Matrix of polychoric correlations

S8. Parallel Analysis and Scree Plots

S9. Unrotated factor matrix

1. **Principal Component Analysis of Protective Behaviors (S1-S3)**

S1. Matrix of polychoric correlations

|  |  |  |  |  |  |  |  |  |  |  |  |  |  |
| --- | --- | --- | --- | --- | --- | --- | --- | --- | --- | --- | --- | --- | --- |
| PREVBEH1 | 1.00 |  |  |  |  |  |  |  |  |  |  |  |  |
| PREVBEH2 | .47 | 1.00 |  |  |  |  |  |  |  |  |  |  |  |
| PREVBEH3 | .43 | .33 | 1.00 |  |  |  |  |  |  |  |  |  |  |
| PREVBEH4 | .35 | .33 | .50 | 1.00 |  |  |  |  |  |  |  |  |  |
| PREVBEH5 | .25 | .20 | .39 | .67 | 1.00 |  |  |  |  |  |  |  |  |
| PREVBEH6 | .48 | .42 | .57 | .63 | .48 | 1.00 |  |  |  |  |  |  |  |
| PREVBEH7 | .37 | .37 | .39 | .34 | .27 | .37 | 1.00 |  |  |  |  |  |  |
| PREVBEH8 | .32 | .35 | .48 | .49 | .29 | .45 | .36 | 1.00 |  |  |  |  |  |
| PREVBEH9 | .43 | .43 | .20 | .35 | .26 | .38 | .47 | .32 | 1.00 |  |  |  |  |
| PREVBEH10 | .47 | .38 | .46 | .35 | .26 | .46 | .63 | .38 | .36 | 1.00 |  |  |  |
| PREVBEH11 | .32 | .22 | .51 | .38 | .25 | .41 | .31 | .47 | .13 | .38 | 1.00 |  |  |
| PREVBEH12 | .43 | .54 | .24 | .29 | .27 | .36 | .41 | .29 | .44 | .31 | .14 | 1.00 |  |
| PREVBEH13 | .30 | .44 | .36 | .30 | .21 | .38 | .32 | .27 | .43 | .34 | .14 | .40 | 1.00 |
|  | PB1 | PB2 | PB3 | PB4 | PB5 | PB6 | PB7 | PB8 | PB9 | PB10 | PB11 | PB12 | PB13 |

S2. Parallel Analysis and Scree Plots


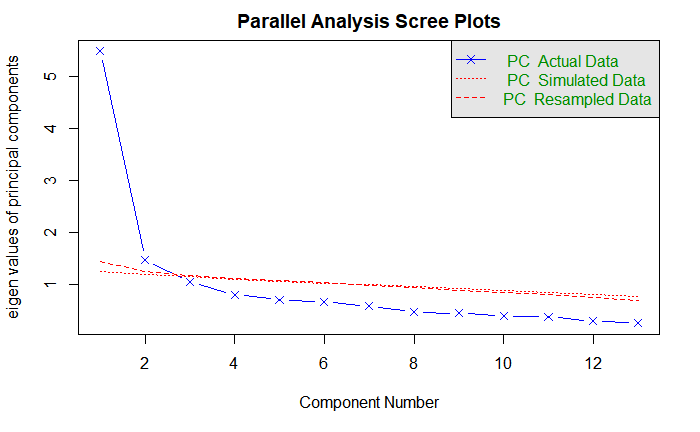


S3. Promax-rotated factor matrix

| **Item** | **Text** | **PC1** | **PC2** | **h2** | **u2** |
| --- | --- | --- | --- | --- | --- |
|  |  |  |  |  |  |
| PREVBEH1 | Wash your hands often | .18 | **.51** | .42 | .58 |
| PREVBEH2 | Use hydroalcoholic solutions for hand washing if made available in public places, gyms, supermarkets, pharmacies and other meeting places | -.04 | **.73** | .49 | .51 |
| PREVBEH3 | Avoid close contact with people you know who suffer from acute respiratory infections | **.74** | .00 | .55 | .45 |
| PREVBEH4 | Avoid hugs and handshakes with your acquaintances | **.80** | -.04 | .60 | .40 |
| PREVBEH5 | Avoid hugs and handshakes with your close relatives | **.62** | -.05 | .35 | .65 |
| PREVBEH6 | Maintaining, in social contacts, an interpersonal distance of at least one meter | **.65** | .17 | .60 | .40 |
| PREVBEH7 | Sneezing and / or coughing in a tissue or elbow, avoiding contact of the hands with respiratory secretions | .16 | **.54** | .42 | .58 |
| PREVBEH8 | Avoid the promiscuous use of bottles and glasses, especially during sports | **.55** | .12 | .40 | .60 |
| PREVBEH9 | Do not touch your eyes, nose and mouth with your hands | -.09 | **.73** | .46 | .54 |
| PREVBEH10 | Cover your mouth and nose if you sneeze or cough | .30 | **.41** | .43 | .57 |
| PREVBEH11 | Do not take antiviral drugs and antibiotics unless prescribed by your doctor | **.67** | -.11 | .36 | .64 |
| PREVBEH12 | Clean the surfaces with chlorine or alcohol-based disinfectants | -.14 | **.77** | .48 | .52 |
| PREVBEH13 | Use the face mask if you suspect you are ill or if you are caring for sick people | .02 | **.56** | .33 | .67 |

Correlation between PC1 and PC2, *r* = .66; h2 = communality; u2 = uniqueness.

Note: PREVBEH Preventive Behavior

1. **Principal Component Analysis of Risk Perceptions (S4-S6)**

S4. Matrix of polychoric correlations

|  |  |  |  |  |  |  |  |  |  |  |
| --- | --- | --- | --- | --- | --- | --- | --- | --- | --- | --- |
| RISKAFF1 | 1.00 |  |  |  |  |  |  |  |  |  |
| RISKAFF2 | .83 | 1.00 |  |  |  |  |  |  |  |  |
| RISKEXP1 | .51 | .46 | 1.00 |  |  |  |  |  |  |  |
| RISKEXP2 | .60 | .60 | .45 | 1.00 |  |  |  |  |  |  |
| RISKPROB | .26 | .27 | .33 | .38 | 1.00 |  |  |  |  |  |
| RISKPERC1 | .65 | .70 | .42 | .45 | .25 | 1.00 |  |  |  |  |
| RISKPERC2 | .51 | .54 | .27 | .34 | .20 | .62 | 1.00 |  |  |  |
| RISKPERC3 | .62 | .64 | .40 | .44 | .23 | .69 | .65 | 1.00 |  |  |
| RISKCOND1 | .33 | .33 | .23 | .38 | .44 | .36 | .37 | .35 | 1.00 |  |
| RISKCOND2 | .29 | .28 | .30 | .28 | .40 | .24 | .25 | .22 | .41 | 1.00 |
|  | AFF1 | AFF2 | EXP1 | EXP2 | PROB | PERC1 | PERC2 | PERC3 | COND1 | COND2 |

S5. Parallel Analysis and Scree Plots


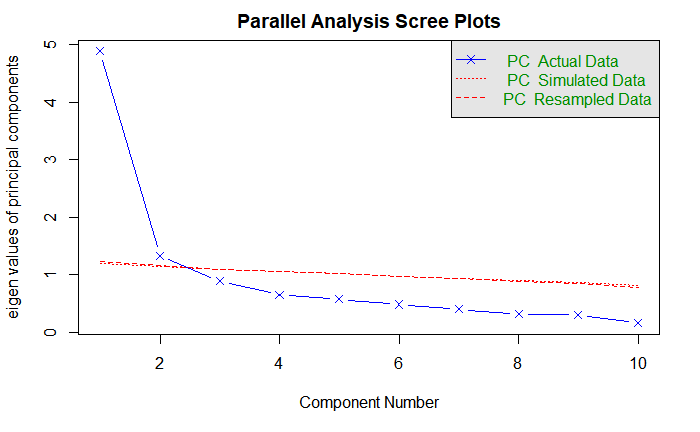


S6. Promax-rotated factor matrix

| **Item** | **Text** | **PC1** | **PC2** | **h2** | **u2** |
| --- | --- | --- | --- | --- | --- |
|  |  |  |  |  |  |
| RISKAFF1 | When you think about coronavirus, to what extent do you feel fearful? | **.84** | .01 | .72 | .28 |
| RISKAFF2 | How worried are you about coronavirus? | **.90** | -.04 | .77 | .23 |
| RISKEXP1 | To what extent do you feel vulnerable to coronavirus? | **.38** | .25 | .32 | .68 |
| RISKEXP2 | When you hear of someone having coronavirus, to what extent your first reaction is ‘that could be me someday’? | **.43** | .31 | .45 | .55 |
| RISKPROB | How likely do you think it is that you will get coronavirus? | -.16 | **.80** | .52 | .48 |
| RISKPERC1 | In general, how risky do you think coronavirus is? | **.87** | -.08 | .67 | .33 |
| RISKPERC2 | In general, how risky do you consider coronavirus to be to Italian society as a whole? | **.69** | -.03 | .45 | .55 |
| RISKPERC3 | How much risk do you believe that coronavirus poses to human health, safety or prosperity? | **.84** | -.08 | .64 | .36 |
| RISKCOND1 | If you did not follow the recommendations to reduce the infection issued by the President of the Council of Ministers, how much do you think it would be likely for you to contract the coronavirus? | .09 | **.56** | .39 | .61 |
| RISKCOND2 | If you continue to adopt the usual lifestyle you have led up to now, how likely would you be to get coronavirus? | -.03 | **.60** | .34 | .66 |
|  |  |  |  |  |  |

Correlation between PC1 and PC2, *r* = .61; h2 = communality; u2 = uniqueness.

Note: RISKAFF = Affective risk perception; RISKPERC = General risk perception; RISKEXP = Experiential risk perception; RISKCOND = Conditional risk perception; RISKPROB = Perceived likelihood.

1. **Principal Component Analysis of Social Norms items (S7-S9)**

S7. Matrix of polychoric correlations

|  |  |  |  |  |  |  |  |
| --- | --- | --- | --- | --- | --- | --- | --- |
| NORMD1 | 1.00 |  |  |  |  |  |  |
| NORMD2 | .77 | 1.00 |  |  |  |  |  |
| NORMD3 | .73 | .68 | 1.00 |  |  |  |  |
| NORMP1 | .66 | .65 | .62 | 1.00 |  |  |  |
| NORMP2 | .65 | .66 | .65 | .68 | 1.00 |  |  |
| NORMP3 | .60 | .58 | .55 | .65 | .61 | 1.00 |  |
| NORMP4 | .36 | .33 | .31 | .46 | .39 | .29 | 1.00 |
|  | D1 | D2 | D3 | P1 | P2 | P3 | P4 |

S8. Parallel Analysis and Scree Plots


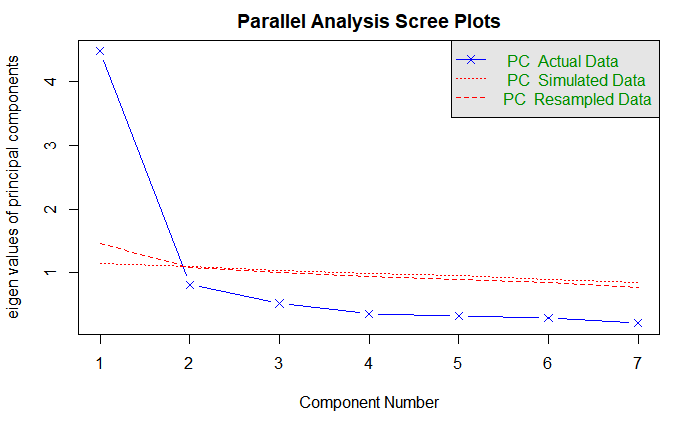


S6. Unrotated factor matrix

| **Item** | **Text** | **PC1** | **h2** | **u2** |
| --- | --- | --- | --- | --- |
|  |  |  |  |  |
| NORMD1 | Most people who are important to me are personally doing something to help reduce the risk from coronavirus. | **.85** | .73 | .27 |
| NORMD2 | Many people I care about do their best to help slow down coronavirus infections. | **.83** | .69 | .31 |
| NORMD3 | People close to me are taking personal actions to reduce the risk of coronavirus. | **.79** | .63 | .37 |
| NORMP1 | In general, I am expected to do my best to help reduce the risk of coronavirus. | **.82** | .68 | .32 |
| NORMP2 | People who are important to me would support me if I decided to help reduce the risk from coronavirus. | **.81** | .66 | .34 |
| NORMP3 | People I respect think I should behave in a way that reduces the risk of coronavirus | **.72** | .52 | .48 |
| NORMP4 (r) | I feel that helping to deal with coronavirus risk is something that is NOT expected from me. | **.44** | .20 | .80 |
|  |  |  |  |  |

(r) reverse scored; h2 = communality; u2 = uniqueness.

Note: NORMP = prescriptive norms; NORMD = Descriptive norms; PREVBEH = protective behavior; AFFATT = Affect.
